# A Role for Frizzled and Their Post-Translational Modifications in the Mammalian Central Nervous System

**DOI:** 10.3389/fcell.2021.692888

**Published:** 2021-08-03

**Authors:** Patricia Pascual-Vargas, Patricia C. Salinas

**Affiliations:** Department of Cell and Developmental Biology, University College London, London, United Kingdom

**Keywords:** post-translational modification, Frizzled receptors, Wnt signalling, CNS connectivity, trafficking

## Abstract

The Wnt pathway is a key signalling cascade that regulates the formation and function of neuronal circuits. The main receptors for Wnts are Frizzled (Fzd) that mediate diverse functions such as neurogenesis, axon guidance, dendritogenesis, synapse formation, and synaptic plasticity. These processes are crucial for the assembly of functional neuronal circuits required for diverse functions ranging from sensory and motor tasks to cognitive performance. Indeed, aberrant Wnt–Fzd signalling has been associated with synaptic defects during development and in neurodegenerative conditions such as Alzheimer’s disease. New studies suggest that the localisation and stability of Fzd receptors play a crucial role in determining Wnt function. Post-translational modifications (PTMs) of Fzd are emerging as an important mechanism that regulates these Wnt receptors. However, only phosphorylation and glycosylation have been described to modulate Fzd function in the central nervous system (CNS). In this review, we discuss the function of Fzd in neuronal circuit connectivity and how PTMs contribute to their function. We also discuss other PTMs, not yet described in the CNS, and how they might modulate the function of Fzd in neuronal connectivity. PTMs could modulate Fzd function by affecting Fzd localisation and stability at the plasma membrane resulting in local effects of Wnt signalling, a feature particularly important in polarised cells such as neurons. Our review highlights the importance of further studies into the role of PTMs on Fzd receptors in the context of neuronal connectivity.

## Introduction

**FIGURE 1:**
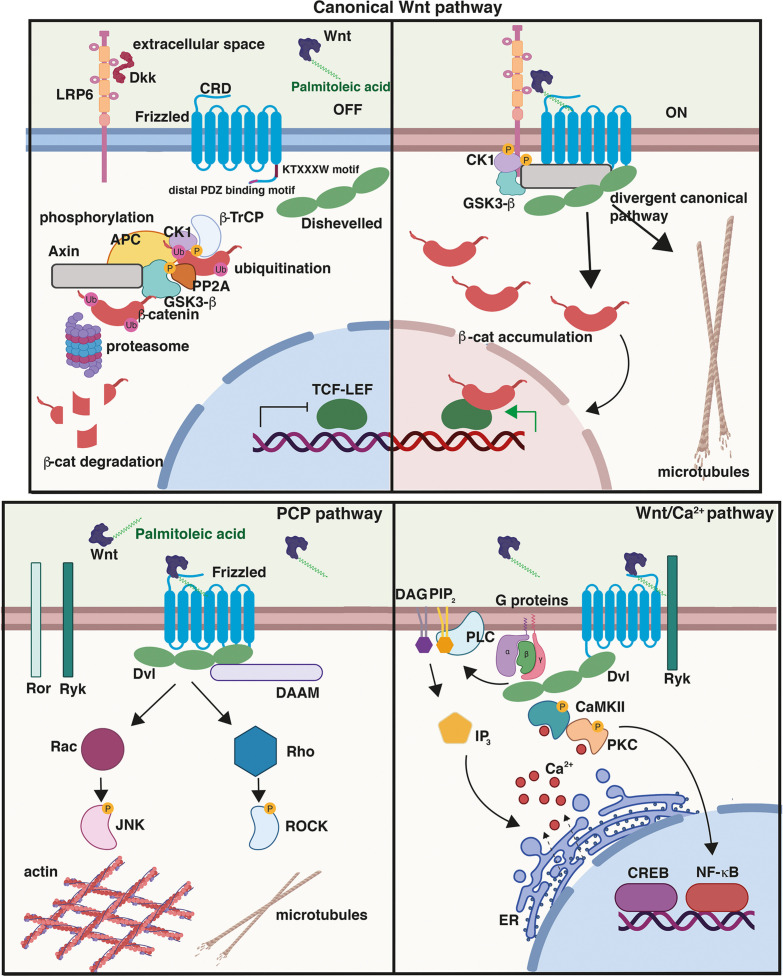
Wnt signalling pathways. (Top) Canonical or Wnt/β-catenin signalling cascade. In the absence of Wnt ligands or in the presence of secreted antagonists like Dkk1, the destruction complex composed of Axin, APC, GSK3β, and β-catenin promotes the ubiquitination and phosphorylation of β-catenin. In the presence of Wnts, Frizzled (Fzd) and co-receptor LRP5/6 dimerise triggering the recruitment of Dvl to the PM and the assembly of signalosome complex; β-catenin is stabilised and then translocates to the nucleus driving the transcription of Wnt target genes through its interaction with TCF/LEF transcription factors. Activation of the divergent canonical/transcription-independent pathway downstream of GSK3β and independent of β-catenin promotes microtubule remodeling which includes axonal remodeling in neurons, cell growth and mitosis in dividing cells, and the maturation of germ cells. Cysteine-rich domain (CRD), conserved KTXXXW Dvl-binding motif, and distal PDZ-binding motifs on Fzd are shown. In the planar cell polarity (PCP) pathway, Wnt binding to Fzd results in the activation of the GTPases Rho and Rac and their effectors ROCK and JNK, respectively, leading to cytoskeletal reorganisation. Activation of the Wnt/Ca^2+^, another β-catenin-independent pathway, results in the release of Ca^2+^ from the endoplasmic reticulum (ER), which activates the protein kinases CAMKII and PKC. This pathway triggers downstream events that include the activation of transcription factors NFκB and CREB and the transcription of downstream regulator genes. Created with BioRender.com.

Wnt secreted proteins are key regulators of neuronal circuit formation and function. Wnts bind to several receptors resulting in the activation of different Wnt signalling pathways. The most common receptors are Frizzled receptors (Fzd), which often interact with co-receptors to initiate different Wnt signalling cascades. Through these receptors, four Wnt signalling cascades can be activated: the canonical Wnt/β-catenin pathway, the Wnt/divergent canonical/transcription-independent pathway, the planar cell polarity (PCP), and the Wnt/calcium pathway (Figure 1) promoting different cellular functions, all of which have been extensively reviewed (Kohn and Moon, 2005; Clevers, 2006; Salinas, 2007; De, 2011; Gray et al., 2011; Devenport, 2014; Acebron and Niehrs, 2016; Nusse and Clevers, 2017).

Frizzled receptors were originally identified in *Drosophila* (Gubb and García-Bellido, 1982), but their role as Wnt receptors was not recognised until 1996 (Bhanot et al., 1996). Functionally, Fzd receptors are critical for the establishment of tissue and cell polarity, embryonic development, regulation of cell proliferation among other processes, and patterning of the central nervous system (CNS) (Huang and Klein, 2004; Wang et al., 2016; Zeng et al., 2018). During postnatal CNS development, Fzd receptors, like their Wnt ligands, play diverse roles including axon guidance, dendritogenesis, and synapse formation (Wang et al., 2016). In the adult nervous system, Wnt–Fzd signalling is required for synaptic plasticity and for synapse maintenance (Inestrosa and Arenas, 2010; Dickins and Salinas, 2013; Oliva et al., 2013a; Purro et al., 2014; Marzo et al., 2016; Buechler and Salinas, 2018; Ferrari et al., 2018; McLeod et al., 2018).

Frizzled receptors belong to the “Frizzled class” within the superfamily of G-protein coupled receptors (GPCRs) (Foord et al., 2005; Schulte and Bryja, 2007; Dijksterhuis et al., 2014) of which 10 Fzd receptors have been identified in mammals, Fzd1–10 (Schulte, 2010). Fzd receptors are 500 to 700 amino acids long and exhibit some characteristics typical of GPCRs: an extracellular N-terminus domain that contains multiple glycosylation sites, followed by seven transmembrane (TM) domains, and an intracellular C-terminus domain, which is subject to post-translational modifications (PTMs) and that interacts with different G proteins (Gα_i_, Gα_q_, and Gα_s_ proteins) (Bhanot et al., 1996; Shulman et al., 1998; Schulte and Bryja, 2007; Nichols et al., 2013). The N-terminus of Fzd receptors contains a fairly conserved cysteine-rich domain (CRD) (Huang and Klein, 2004; Wang Y. et al., 2006), characterised by a hydrophobic cavity required for binding to the palmitoleate moiety present on Wnt ligands (Figure 1; Janda et al., 2012; DeBruine et al., 2017; Nile and Hannoush, 2018). In contrast, the intracellular C-terminus is highly variable between different Fzd receptors, except for a highly conserved KTXXXW motif required for binding to the scaffold protein Dishevelled (Dvl), a critical component of all Wnt cascades (Wang H. et al., 2006; Sharma et al., 2018). These findings suggest that although the C-terminus and PTMs of Fzd receptors may vary, these receptors require Dvl to activate Wnt signalling.

Frizzled–Wnt signalling is important for maintaining a healthy nervous system. Indeed, deficiency in Fzd receptors leads to neurodevelopmental defects (Wang et al., 2016). For example, Fzd9 deficiency is responsible for some of the aspects of the multisystem developmental disorder Williams–Beuren (Ranheim et al., 2005; Zhao et al., 2005). Furthermore, impaired Wnt signalling has been linked to neurodegenerative diseases such as Alzheimer’s disease (Purro et al., 2012; Sellers et al., 2018), and downregulation of *FZD2* and *FZD3* has been observed in the aging brain (Folke et al., 2019). This raises the important question: how is Fzd function regulated in health and disease? Fzd receptors are essential for Wnt signalling activation and their function is modulated by PTMs such as glycosylation, phosphorylation, and ubiquitination, all of which can affect trafficking, localisation, and their ability to signal. However, these PTMs are largely understudied in the nervous system. Here, we review the current understanding of Fzd PTMs and how those described in the CNS affect Fzd function during postnatal development of the mammalian CNS. For PTMs described outside the CNS, we discuss how they could play a role in Fzd function in neuronal connectivity.

## Function of Frizzled Receptors in the Mammalian CNS

Frizzled receptors are crucial Wnt receptors that mediate diverse functions in neurons including neurogenesis, axon guidance, dendritogenesis, synapse formation, and synaptic plasticity (Sahores et al., 2010; Budnik and Salinas, 2011; Oliva et al., 2013b; Ferrari et al., 2018; McLeod et al., 2018). Fzd receptors are enriched at specific subcellular compartments (Wu et al., 2004), suggesting that specific subcellular localisation of Fzd could allow the activation of the Wnt pathway in specific compartments and not the entire cell. This feature would be crucial in polarised cells such as neurons, during axon guidance and synapse assembly. Indeed, local activation of Wnt signalling *via* CAMKII is critical to regulate spine growth and synaptic strength at dendritic spines (Ciani et al., 2011). Here, we focus on Fzd receptors involved in CNS connectivity.

**FIGURE 2:**
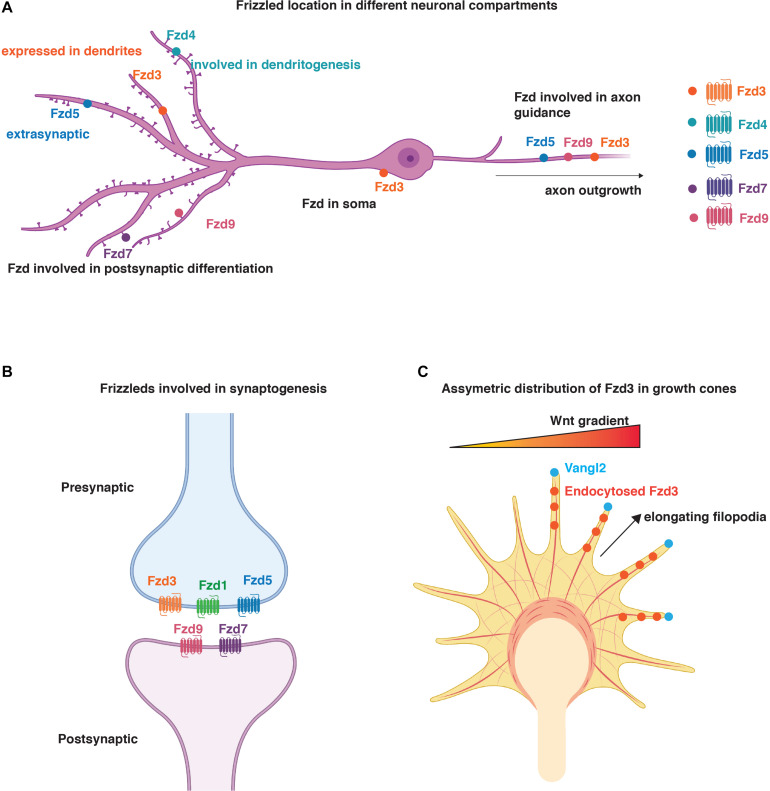
Distribution and function of Fzd in neurons. **(A)** Schematic representation of localisation of Fzd receptors to different neuronal compartments. Fzd3 is localised to the soma, axons, and dendrites. Fzd4, Fzd7, and Fzd9 are present in dendrites and promote dendritogenesis and/or synaptogenesis. Fzd3, Fzd5, and Fzd9 play a role in axon outgrowth. Fzd4 is also involved in dendritogenesis. Fzd5 is present in axons where it regulates synapse formation. It is also present in dendrites but its function in this neuronal compartment is unknown. **(B)** Schematic representation of Fzd involved in synaptogenesis. Fzd3, Fzd1, and Fzd5 are presynaptically localised, whereas Fzd9 and Fzd7 are present postsynaptically. **(C)** Representation of asymmetric distribution of Fzd3 in growth cones due to the asymmetric localisation of Vangl2 at elongating filopodia. Here, Vangl2 promotes Fzd3 endocytosis and PCP signalling in certain filopodia to ensure asymmetric signalling for growth cone steering in response to Wnt gradients (Onishi et al., 2014). **(A–C)** Created with BioRender.com.

Axon guidance is a crucial process that permits the navigation of axons to their appropriate targets during development (Salinas, 2012; Stoeckli, 2018). This process is followed by the terminal remodeling of axons and growth cones that allow the subsequent assembly of the synapses (Salinas, 2012). Two receptors, Fzd5 and Fzd9, are present on axonal growth cones (Shah et al., 2009; Slater et al., 2013). Fzd9 is selectively expressed in the developing and adult hippocampus, where it localises to dendrites and efferent axons postnatally (Table 1; Zhao and Pleasure, 2004). In contrast, Fzd5 is expressed at the peak of synaptogenesis in the mouse hippocampus, with expression increasing postnatally (Sahores et al., 2010). In cultured rat hippocampal neurons, Fzd9 is present along the axon and axonal growth cones during the early stages of their development (Shah et al., 2009; Varela-Nallar et al., 2012), whereas Fzd5 is distributed exclusively on axonal growth cones during development up to the stage of dendritic outgrowth (Varela-Nallar et al., 2012; Slater et al., 2013), but in both axons and dendrites from neuronal maturation stage (Sahores et al., 2010; Figure 2A). This suggests that Fzd5 could play a role in the initial establishment of neuronal polarity and in the morphogenesis of neuronal processes and that its function may differ in dendrites versus axons (Table 1). Indeed, loss and gain-of-function experiments showed that Fzd5 is required for neuronal polarity and neurite growth (Slater et al., 2013). Studies on another Fzd, Fzd3, demonstrated that this receptor is required *in vivo* for axon growth and guidance in the forebrain, cranial and spinal motor neurons, sensory neurons, and the sympathetic nervous system, mainly through activation of the PCP signalling pathway (Wang et al., 2002, 2016; Lyuksyutova et al., 2003; Fenstermaker et al., 2010; Armstrong et al., 2011; Hua et al., 2013, 2014; Schafer et al., 2015; Feng et al., 2016; Ghimire and Deans, 2019). However, its role in postnatal development is less known. Overall, these findings show that specific enrichment of Fzd5, Fzd9, and Fzd3 in growth cones is required for axonal outgrowth in different brain regions.

**TABLE 1:** Frizzled (Fzd) receptors in the nervous system: localisation, function, and post-translational modifications (PTMs).

Fzd	Localisation	Function	PTMs (CNS)	PTMs (outside CNS)	Major interacting partners	References
Fzd1	Presynaptic	Presynaptic differentiation			Wnt3a	Varela-Nallar et al. (2009)
Fzd3	Soma, dendrites, axons, and synapses	*In vivo* for axon growth and guidance, synaptogenesis in dendrites, presynaptic differentiation	Phosphorylation: • Hyperphosphorylation required for PCP signalling and growth cone guidance *in vivo* • Hyperphosphorylation in presynaptic fraction Glycosylation: • N42 in CRD domain and N356 in second extracellular loop • Shisa2 inhibits glycosylation of Fzd3, reducing surface levels		PCP signalling components: Celsr3, Vangl2	Wang et al. (2002, 2016), Lyuksyutova et al. (2003); Fenstermaker et al. (2010), Armstrong et al. (2011); Shafer et al. (2011), Hua et al. (2013, 2014), Onishi et al. (2013); Schafer et al. (2015), Feng et al. (2016); Thakar et al. (2017), Ghimire and Deans (2019)
Fzd4	Dendrites	Activity-independent dendrite morphogenesis			Wnt5a	Bian et al. (2015)
Fzd5	Axons, dendritic shaft	Initial establishment of axonal polarity, presynaptic differentiation		• Ubiquitination by ZNRF3 and RNF43 which results in receptor endocytosis and degradation • Deubiquitination by USP6, maintains receptor at PM	Wnt7a	Sahores et al. (2010); Hao et al. (2012), Koo et al. (2012); Varela-Nallar et al. (2012), Slater et al. (2013); Madan et al. (2016), McLeod et al. (2018)
Fzd7	Dendritic growth cones, postsynapse	Dendritogenesis, postsynaptic differentiation		Inhibition of N-linked glycosylation by Shisa in the ER suppresses maturation and trafficking of Fzd to PM in HEK293	Wnt7a	Yamamoto et al. (2005); Varela-Nallar et al. (2012), Ferrari et al. (2018); McLeod et al. (2018)
Fzd9	Axon, dendrites	Axon guidance, postsynaptic assembly			Wnt5a, non-canonical Wnt signalling pathway	Shah et al. (2009); Varela-Nallar et al. (2012), Ramírez et al. (2016)

Dendritic morphogenesis is essential for the formation of functional neuronal networks (Prigge and Kay, 2018; Lefebvre, 2021). Fzd4 is localised in dendrites where it mediates activity-independent dendrite morphogenesis downstream of Wnt5a during postnatal development, as demonstrated by loss and gain-of-function experiments in cultured hippocampal neurons (Table 1; Bian et al., 2015). Fzd4 mRNA expression levels increase during the first two postnatal weeks in the mouse hippocampus and cerebral cortex, concomitantly with *Wnt5a* expression (Bian et al., 2015). Interestingly, signalling *via* Fzd4 is through its less conserved distal PDZ-binding motif rather than *via* the conserved motif to which Dvl1 binds (Wong et al., 2003; Bian et al., 2015). Although the function of Dvl1 has not been ruled out completely, the data suggest a primarily Dvl1-independent mechanism (Bian et al., 2015). This is of particular importance as Dvl is crucial for integrating and coordinating the activation of all Wnt signalling cascades (Sharma et al., 2018). In addition, Fzd4 is important for other brain functions. *In vivo* studies suggest that Fzd4 is important for the maintenance of blood–brain barrier function and plasticity in the mature CNS vascular structure (Wang et al., 2012).

Fzd7 is another receptor that plays an important role in dendritogenesis (Table 1 and Figure 2A). In the hippocampus, Fzd7 expression increases from birth, reaching its peak in the adult (Ferrari et al., 2018). Endogenous Fzd7 localises along the neurite shaft and at dendritic growth cones (Ferrari et al., 2018). Loss and gain-of-function studies in cultured hippocampal neurons and loss-of-function *in vivo* showed that Fzd7 functions as a receptor for Wnt7b-mediated dendritic growth and complexity *via* Dvl1, CAMKII, and JNK (Ferrari et al., 2018). Therefore, Fzd7 promotes dendritogenesis *via* two non-canonical Wnt pathways (Ferrari et al., 2018).

Synapse formation is a complex process that requires the coordinated assembly of thousands of proteins at both sides of the synapse. Fzd receptors such as Fzd1, Fzd3, Fzd5, Fzd7, and Fzd9 play an important role in synaptogenesis (Varela-Nallar et al., 2009; Sahores et al., 2010; Schafer et al., 2015; Ramírez et al., 2016; McLeod et al., 2018). In particular, Fzd1, Fzd3, and Fzd5 regulate presynaptic differentiation, whereas Fzd7 and Fzd9 are important for postsynaptic assembly (Table 1 and Figure 2B). These findings highlight the idea of local signalling, where enrichment of different Fzd receptors in distinct cellular compartments results in specific outcomes such as pre- or postsynaptic development.

Fzd1 is highly expressed in the hippocampus during postnatal development (Shimogori et al., 2004; Lein et al., 2007; Varela-Nallar et al., 2009; Mardones et al., 2016). Wnt3a ligand through Fzd1 regulates presynaptic differentiation and function as both the overexpression of Fzd1 and treatment with Wnt3a increase the number of bassoon puncta, a function that is blocked by exposure to a peptide containing the CRD domain of Fzd1. Notably, Fzd1 is present in synaptosome fractions from adult rat brains, suggesting a role in mature synaptic function (Varela-Nallar et al., 2009), but this has not been demonstrated.

In cultured hippocampal neurons, Fzd3 is expressed in the cell bodies, dendrites, and axons (Figure 2A) and co-localises with the presynaptic marker Vglut1 (Davis et al., 2008). Consistently, *in vivo* studies demonstrate that Fzd3 is localised presynaptically at glutamatergic synapses where it interacts with Cadherin EGF LAG seven-pass G-type receptor 3 (Celsr3), a key component of the PCP pathway, to promote synapse formation in the postnatal hippocampus (Thakar et al., 2017). These results suggest that Fzd3 interacts with PCP signalling components at the presynaptic side to promote glutamatergic synapse formation.

In addition to its role in neuronal polarity and neurite outgrowth (Varela-Nallar et al., 2012; Slater et al., 2013), Fzd5 also plays a role in presynaptic assembly (Table 1). Studies using loss and gain-of-function studies in cultured hippocampal neurons demonstrated that Fzd5 is a presynaptic receptor for Wnt7a (Sahores et al., 2010). Fzd5 KD by expression of shRNAs or by acute Fzd5 loss-of-function achieved by using the soluble Fzd5-CRD domain that binds Wnts blocks Wnt7a-mediated synaptogenesis in cultured hippocampal neurons. In contrast, Wnt7a treatment or gain-of-function of Fzd5 induces presynaptic assembly (Sahores et al., 2010). Notably, high-frequency stimulation (HFS), a well-established paradigm that induces long-term potentiation and the formation of synapses (Bolshakov et al., 1997; Bozdagi et al., 2000), increases surface levels of Fzd5. In contrast, blockade of Fzd5 function with CRD domain during HFS prevents the localisation of Fzd5 to cell surface and to synapses. Importantly, the CRD domain of Fzd5 fully blocks HFS-induced synaptogenesis (Sahores et al., 2010). The results demonstrate that Fzd5 is required for activity-mediated synapse formation.

Fzd5 is also present along the dendritic shaft but is not enriched at dendritic spines (McLeod et al., 2018; Figure 2A). This finding raises the question of the role of Fzd5 in dendrites and how Fzd receptors are trafficked to those specific cellular locations. Interestingly, *Fzd5* is also expressed in the retina, the hypothalamus, and the parafascicular nucleus (PFN) of the thalamus (Shimogori et al., 2004; Liu et al., 2008), and studies of Fzd5-deficient mice revealed that Fzd5 is required for the survival of adult PFN neurons but not for their development (Liu et al., 2008). In summary, Fzd5 plays diverse roles in the postnatal CNS, but its potential role in dendrites requires further studies.

Two other Fzd receptors (Fzd7 and Fzd9) are expressed in the hippocampus. Their expression increases from birth, reaching its peak in the adult (Shimogori et al., 2004; Varela-Nallar et al., 2012; Ferrari et al., 2018). In mature neurons, Fzd7 is enriched in the postsynaptic fraction and localises to dendritic spines (Table 1; McLeod et al., 2018). Loss and gain-of-function studies of Fzd7 as well as acute blockade of endogenous Wnts in cultured hippocampal neurons showed that Wnt7a–Fzd7 postsynaptic signalling mediates LTP-dependent spine plasticity by promoting synaptic AMPA receptor localisation *via* CAMKII, PKA, and ERK cascades (McLeod et al., 2018). Thus, Fzd7 is required for Wnt7a/b-mediated dendritic development and for structural and functional plasticity of synapses (McLeod et al., 2018).

Fzd9 is highly enriched in postsynaptic synaptosome preparations from adult rat brains. Importantly, loss-of-function studies demonstrate that Fzd9 is required for Wnt5a-mediated increase in dendritic spine density in cultured hippocampal neurons (Table 1; Ramírez et al., 2016). Mechanistically, Fzd9 interacts with heterotrimeric G proteins resulting in the activation of non-canonical Wnt signalling pathways including CAMKII, JNK, and protein kinase C (PKC) (Ramírez et al., 2016). However, the *in vivo* role for Wnt5a/Fzd9 signalling has not been examined.

## How PTMs Determine the Function of Frizzled Receptors

Post-translational modifications are biochemical modifications that are incorporated onto one or more amino acids after protein synthesis. PTMs vary greatly, but they all modulate the biochemical properties of proteins beyond that conferred by amino acids alone. PTMs are master regulators of protein trafficking, subcellular localisation, and function. There are different types of PTMs ranging from the covalent attachment of proteins or functional groups to their proteolytic cleavage (Mann and Jensen, 2003; Kannicht and Fuchs, 2008; Khoury et al., 2011; Vidal, 2011; Millar et al., 2019). The most common forms of PTM are N-linked glycosylation and phosphorylation (Khoury et al., 2011).

Wnts are post-translationally modified proteins. As secreted proteins, they are glycosylated but have a unique PTM as they are palmitoyleolated by a membrane-bound O-acyltransferase called Porcupine in the ER, which adds palmitoleic acid to serine residues on the Wnt protein (Hausmann et al., 2007; Nile and Hannoush, 2018). This modification regulates Wnt secretion and their interaction with Frizzled receptors (Kakugawa et al., 2015). Palmitoyleolation is a reversible PTM as Wnts can be de-palmitoyleolated by the de-acylase protein Notum in the extracellular space, resulting in the inhibition of Wnt signalling (Kakugawa et al., 2015). In turn, Fzd receptors are also PTM by glycosylation, ubiquitination, and phosphorylation that modify their localisation and function. To date, phosphorylation is the most described PTM on Fzd receptors in the CNS, followed by glycosylation.

The distribution of Fzd at the PM confers spatio-temporal dynamics of signalling activation (Huang and Klein, 2004). For example, Fzd5 is enriched presynaptically (Sahores et al., 2010), whereas Fzd9 is enriched postsynaptically (Ramírez et al., 2016), raising the question: what controls the trafficking and retention of Fzd receptors at these specific PM locations? A possible mechanism could be through PTMs. Indeed, Fzd receptors are phosphorylated, an important modification that regulates their function during axon guidance (Shafer et al., 2011; Onishi et al., 2013). Fzd receptors have canonical motifs for phosphorylation by protein kinase A (PKA) and PKC and casein kinase II on their C-terminus (Wang and Malbon, 2004). As Fzd receptors have highly variable C-terminal domains, it is possible that not all Fzd receptors will be modified by phosphorylation and could be regulated in different ways. For example, in mammalian systems, Fzd6 has been reported to be phosphorylated on pSer-648 by casein kinase I d in epithelial cells (Strakova et al., 2018), whereas Dvl-dependent phosphorylation of Fzd3 requires Ser-576 (Yanfeng et al., 2006). Additional sites for the phosphorylation of Fzd3 were also identified: Ser-508, Thr-541, Thr-562, Ser-587, Ser-624, and Ser-636, all of which reduced Dvl-induced phosphorylation when mutated to alanine (Yanfeng et al., 2006).

A particular example of the regulation of Fzd phosphorylation is through Celsr3, an essential component of the PCP pathway, which regulates Fzd3 phosphorylation. Mice lacking Celsr exhibit impaired PCP signalling and hyperphosphorylated Fzd3 (Wang and Nathans, 2007; Onishi et al., 2013). In addition, a recent study showed that both loss-of-function of leucine-rich repeat kinase 2 (LRRK2) and gain-of-function of mutated LRRK2 lacking kinase activity showed anterior–posterior guidance errors after midline crossing *in vivo* (Onishi et al., 2020). Furthermore, they identified LRRK2 as a new protein which both directly phosphorylates Fzd3 on threonine 598 (T598) and indirectly promotes Dvl1-induced Fzd3 hyperphosphorylation by acting as a scaffold and recruiting other kinases (Onishi et al., 2020). Overall, hypo- or non-phosphorylated Fzd3 is required for PCP signalling and for growth cone guidance *in vivo* (Table 1; Shafer et al., 2011; Onishi et al., 2013, 2020).

Studies also showed a correlation between the level of Fzd3 phosphorylation and endocytosis. Activation of the PCP pathway requires Fzd receptor endocytosis (Yu et al., 2007; Sato et al., 2010). Consistent with this, overexpression of Dvl1 and a mutant of Fzd3, whose phosphorylation cannot be induced by Dvl1, promotes Fzd3 internalisation and the consequent increase in PCP signalling in dissociated commissural neurons (Shafer et al., 2011; Onishi et al., 2013). In addition, stimulation with recombinant Wnt5a results in Fzd3 endocytosis at growth cones of dissociated commissural neurons, where Dvl2 and atypical PKC (aPKC) inhibit Dvl1-induced hyperphosphorylation of Fzd3. This process leads to Fzd3 endocytosis and the consequent increase in PCP signalling (Onishi et al., 2013). Together, these data show that Dvl1-mediated hyperphosphorylated Fzd3 is maintained at the PM and inhibits PCP signalling, whereas hypo- or non-phosphorylated Fzd3 is internalised resulting in the activation of the PCP cascade (Table 1). Thus, phosphorylation is an important PTM that regulates the localisation of Fzd3 at the PM and its ability to signal. In contrast to Fzd3, the role of phosphorylation on other Fzd remains to be determined.

Loss and gain-of-function experiments led to the conclusion that another core PCP pathway component Vangl2 (Van Gogh2), a four pass transmembrane protein, also inhibits Dvl1-mediated Fzd3 hyperphosphorylation and promotes its endocytosis resulting in PCP signalling (Shafer et al., 2011). Vangl2 is localised predominantly at the PM and is enriched at the tips of filopodia and sites where filopodia emerge in commissural axon growth cones. In addition, when Dvl1 and Fzd3 are co-expressed, they target each other to the plasma membrane of growth cones (Shafer et al., 2011). The antagonistic actions of Dvl1 and Vangl2 on Fzd3 phosphorylation allow the sharpening of PCP signalling to occur locally on tips of filopodia to sense directional cues by Wnts causing the turning of growth cones (Shafer et al., 2011).

Another interesting feature is the asymmetric localisation of Wnt components in neurons. Vangl2 is localised asymmetrically to the tip of elongating filopodia as opposed to those that are shrinking, suggesting that Vangl2 promotes Fzd3 endocytosis and PCP signalling in some filopodia to ensure asymmetric response to Wnts and, consequently, signalling for growth cone steering (Onishi et al., 2014; Figure 2C). Whether the asymmetric localisation of Fzd3 is due to changes in PTM remains to be determined.

Vangl2 also antagonises Celsr3 during glutamatergic synapse formation as Vangl2 inhibits synapse assembly, whereas Celsr3 promotes synapse assembly (Thakar et al., 2017). Analysis of the presence of PCP components at synaptic membrane fractions (SMF) and postsynaptic density (PSD) demonstrates that Celsr3 and Celsr2 are present in both fractions. In addition, Dvl1 is enriched in SMF, whereas Dvl2 and Vangl2 are enriched in PSD fraction (Thakar et al., 2017). Interestingly, the hyperphosphorylated form of Fzd3 is more abundant in the SMF than the PSD, and the unphosphorylated form of this receptor is enriched in the PSD fraction. These findings are consistent with the data showing that Fzd3 is hyperphosphorylated in a Dvl1-mediated manner, a process which is inhibited by Dvl2 and Vangl2 (Shafer et al., 2011; Onishi et al., 2013). Overall, these data suggest that PCP components are asymmetrically localised in glutamatergic synapses (Thakar et al., 2017) to mediate local effects on PCP signalling as observed by hyperphosphorylated and unphosphorylated forms of Fzd3.

Asymmetric distribution of Fzd3, which is closely related to Fzd6 (Schulte, 2010), has been observed outside the CNS. In epithelial cells of the fallopian tube, phosphorylated Fzd6 (pSer-648 by casein kinase I d) is predominantly localised to the apical side compared with total Fzd6, which is evenly distributed on both the apical and basal plasma membrane of these epithelial cells (Strakova et al., 2018). This could suggest that asymmetric phosphorylation rather than asymmetric distribution of Fzd6 leads to polarised signalling (Strakova et al., 2018). Overall, these studies demonstrate that phosphorylation is an important PTM, which affects the function of Fzd both in epithelial cells and the CNS and is responsible for polarised and, therefore, local signalling.

**FIGURE 3:**
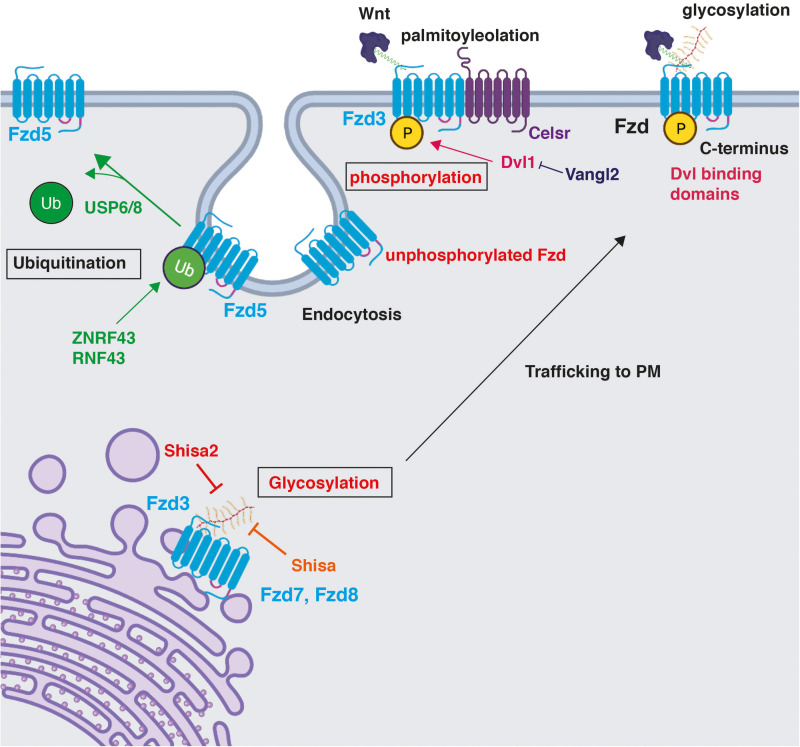
Post-translational modifications on Frizzled receptors and their impact on Fzd function. Fzd receptors are post-translationally modified. Phosphorylation and glycosylation are shown in red as described in the CNS. Phosphorylation maintains Fzd at the PM (Shafer et al., 2011; Onishi et al., 2013). A particular example of the regulation of Fzd phosphorylation is through Celsr3, an essential component of the PCP pathway. Dvl1 promotes Fzd3 hyperphosphorylation, which is inhibited by Vangl2 (Shafer et al., 2011). Glycosylation occurs on the N-terminus in the ER and promotes trafficking of Fzd7 and Fzd8 to the plasma membrane, a process that is inhibited by the ER-resident protein Shisa in HEK293 (Yamamoto et al., 2005), and by Shisa2 in rat commissural neurons (Onishi and Zou, 2017). Ubiquitination by ZNRF3 and RNF43 occurs on multiple lysine residues in cytoplasmic loops and results in Fzd5 internalisation and degradation (Hao et al., 2012; Koo et al., 2012; Moffat et al., 2014). USP6/8 deubiquitinate Fzd5, stabilising the membrane pool of Fzd5 and promoting its recycling to the PM (Madan et al., 2016). Created with BioRender.com.

Although phosphorylation is the best understood PTM for Fzd in the CNS, glycosylation of Fzd has also been described in rodent commissural axon growth cones (Onishi and Zou, 2017). Fzd receptors were first identified to be glycosylated at the N-terminus in the ER in HEK293 cells suggesting that this PTM might be important for Fzd maturation and trafficking to the PM (Yamamoto et al., 2005; Figure 3). Shisa, an ER-resident protein, interacts with immature Fzd protein in the ER, preventing further processing of N-linked glycosylation and suppressing its maturation and trafficking to the cell surface in HEK293 (Yamamoto et al., 2005). Therefore, Shisa inhibits Wnt signalling. The role of Shisa has been demonstrated for Fzd7 and Fzd8; however, all Fzd receptors are predicted to be N-glycosylated at a conserved YNTxT motif (Table 1; MacDonald and He, 2012). Indeed, Shisa2 was later on shown to inhibit Fzd3 glycosylation at two sites (N42 in the CRD domain and N356 in the second extracellular loop) and, consequently, Fzd3 cell surface presentation in HEK293 (Onishi and Zou, 2017). Shisa2 knockdown in commissural neurons was also shown to increase Fzd3 protein on the surface of growth cones, resulting in precocious anterior turning of commissural axons before or during midline crossing (Table 1; Onishi and Zou, 2017). In addition, Shh-Smoothened signalling activates PCP signalling in commissural neurons by inhibiting Shisa2 and, therefore, resulting in Fzd3 trafficking to the membrane and activation of PCP signalling (Onishi and Zou, 2017). Further studies are required to establish the precise role of Shisa on other Fzd receptors in different cellular contexts in the CNS. For example, at the synapse, Shisa9, the first Shisa to be identified in mice, plays an important role in AMPA receptor (AMPAR) desensitisation (von Engelhardt et al., 2010). Another isoform, Shisa7, is associated with AMPAR and regulates synaptic function (Schmitz et al., 2017). In contrast, Shisa6 traps AMPAR at postsynaptic sites (Klaassen et al., 2016). The presence of different Shisa proteins in the brain raises the question: which Shisa isoforms regulate Fzd localisation and function in the mammalian CNS? Is Shisa2 specific to Fzd3 in axonal growth cones? What is the relationship of Shisa with Fzd at the synapse and other neuronal compartments?

Another PTM that regulates Fzd receptor function, but has not been described in the CNS, is ubiquitination, where cycles of ubiquitination/deubiquitination control the degradation of Fzd receptors and their recycling to the PM (Hao et al., 2012; Koo et al., 2012; Moffat et al., 2014). Fzd can be multi-monoubiquitinated on lysine residues, located in their cytoplasmic loops between different transmembrane domains, by the highly related and membrane-localised RING E3 ligases Zinc and acid finger protein 3 (ZNRF3) and ring finger protein 43 (RNF43) (Hao et al., 2012; Koo et al., 2012). Studies in HEK293T cells show that a Fzd5 mutant, which cannot be ubiquitinated as all cytosolic residues are mutated to arginine, is resistant to RNF43-mediated internalisation, suggesting that ubiquitination plays an important role in Fzd internalisation and, therefore, has the ability to detect Wnt ligands (Table 1; Koo et al., 2012). Indeed, ubiquitination of Fzd reduces their levels at the PM resulting in the downregulation of Wnt signalling by promoting Fzd endocytosis and lysosomal degradation (Hao et al., 2012; Koo et al., 2012; Moffat et al., 2014; Figure 3). Interestingly, ubiquitination by ZNRF3 and RNF43 has also been shown to regulate surface levels of the Wnt co-receptor LRP5/6 in the same way as Fzd (Hao et al., 2012; Koo et al., 2012). Wnt binding to Fzd and co-receptor LRP5/6 induces the dimerisation of these two receptors activating signalling *via* the β-catenin canonical pathway (Bilic et al., 2007; Komiya and Habas, 2008; Feng and Gao, 2015; Nusse and Clevers, 2017). Therefore, this raises the question of whether Fzd and co-receptors are regulated in the same manner and whether PTMs on Fzd influence their interaction with co-receptors. Future research will shed new light into this.

R-spondins are secreted glycoproteins and are effective Wnt agonists that bind to ZNRF3 forming a trimeric complex between R-spondin, the R-spondin receptor LGR4, and ZNRF3, which results in the membrane clearance of ZNRF3 (Hao et al., 2012). Therefore, R-spondins inhibit ZNRF3-dependent ubiquitination of Fzd receptors, thus potentiating Wnt signalling (Hao et al., 2012). Double knockout of ZNFR3 and RNF43 induces intestinal adenoma, a Wnt-dependent cancer, likely as a result of increased Fzd receptors at the cell surface (Koo et al., 2012). Indeed, loss-of-function mutations of ZNFR3/RNF42 have been observed in many other types of cancers (Koo et al., 2012; Hao et al., 2016; Katoh and Katoh, 2017). In contrast, Fzd receptors are deubiquitinated by UBPY/ubiquitin-specific protease 6 and 8 (USP6 and USP8). Gain and loss-of-function studies showed that USP6 stabilises the membrane pool of Fzd5 (Table 1; Madan et al., 2016) and USP8 promotes the recycling of Fzd receptors to the PM, increasing their cell surface localisation and therefore enhancing Wnt signalling both in mammalian cells and *Drosophila* wing (Mukai et al., 2010). USP6 has also been shown to regulate cell surface abundance of LRP6 as expression of wild-type USP6 increases surface levels of LRP6. However, it is unclear whether it directly deubiquitylates LRP6 (Madan et al., 2016). Interestingly, the Wnt antagonist Dickkopf-1 (DKK1) or LRP5/6 siRNA blocked USP6-induced Wnt signalling, suggesting that a Wnt-ligand receptor complex is required for USP6 function (Madan et al., 2016). These data support the view that ubiquitination/deubiquitination cycles are crucial in regulating surface levels of Fzd and co-receptors LRP5/6 and, therefore, Wnt signalling.

## Discussion

Increasing evidence suggests that PTMs of Frizzled receptors play important roles in their function and cellular localisation. However, only phosphorylation and glycosylation have been described to modulate Fzd function in the central nervous system (CNS). Here, we have focussed our attention on the specific cellular localisations of Fzd in the mammalian CNS and how PTMs contribute to their function in the CNS. The exact mechanisms for these modifications remain poorly understood.

Currently, phosphorylation is the most understood PTM of Fzd in the CNS. This PTM regulates Fzd3 localisation at the PM during growth cone guidance and glutamatergic synapse formation, where asymmetric distribution of unphosphorylated/phosphorylated Fzd3 has been observed alongside asymmetric distribution of components regulating Fzd3 phosphorylation (Shafer et al., 2011; Onishi et al., 2013; Onishi and Zou, 2017; Thakar et al., 2017). Thus, asymmetric distribution or asymmetric phosphorylation of Fzd is important for local signalling.

Glycosylation has also been shown to be important in controlling surface levels of Fzd both in HEK293 cells (Fzd7 and Fzd8) and in the CNS (Fzd3). Inhibition of glycosylation by Shisa proteins in both systems decreased Fzd receptors at the PM and, consequently, dampened Wnt signalling (Yamamoto et al., 2005; Onishi et al., 2020). As many key proteins involved in synaptic transmission are N-glycosylated, this PTM on Fz receptors could be an important regulator of neurotransmitter release, excitability and synaptic potentiation (Scott and Panin, 2014). Further studies are required to establish the precise role of different Shisa proteins on Fzd receptors in the context of neuronal connectivity.

On the other hand, ubiquitination of Fzd has only been described in cell lines. The role of ubiquitination/deubiquitination cycles on Fzd is better understood as these processes control the degradation of Fzd receptors and their recycling to the PM in cell lines (Hao et al., 2012; Koo et al., 2012; Moffat et al., 2014). Given the data in heterologous cells and how important glycosylation and ubiquitination/deubiquitination regulate surface localisation of receptors such as AMPAR and GABAR in the CNS (von Engelhardt et al., 2010; Klaassen et al., 2016; Schmitz et al., 2017), it is likely that these PTMs regulate the trafficking, localisation, and function of Fzd in the CNS.

By modulating the stability of Fzd at the PM, PTMs modulate their ability to signal. For example, PTMs could influence the interaction of Fzd with their co-receptors such as LRP5/6 thereby affecting canonical Wnt signalling or other pathways. Interestingly, LRP5/6 surface levels are modulated by ubiquitination in the same was as Fzd (Koo et al., 2012; Hao et al., 2016), and deubiquitination by USP6 requires a Wnt–ligand-engaged Fzd–LRP5/6 receptor complex (Madan et al., 2016). Similarly, PTM could regulate the interaction between Fzd and another co-receptor such as Ror or Ryk, influencing the PCP pathway. This raises the question of whether PTM of Fzd receptors and their co-receptors are coordinated and whether certain PTMs depend on Wnt ligand interaction.

In summary, Fzd are post-translationally modified by phosphorylation, glycosylation, and ubiquitination/deubiquitination. In the CNS, however, although phosphorylation of Fzd is the best understood PTM, it has only been described for Fzd3. Indeed, phosphorylation plays a role in the asymmetric distribution of Fzd3 receptors during axon guidance and at the synapse. However, whether other Fzd receptors are regulated by phosphorylation and how this PTM will affect their function remains to be determined. As phosphorylation occurs on the highly variable C-terminal of Fzd receptors, this PTM is likely to vary between Fzd receptors. In contrast, although glycosylation has been demonstrated to be important only for Fzd3 at the cell surface during axon guidance so far, given that it has been shown to regulate Fzd7 and Fzd8 in the same manner in HEK293 cells, combined with the fact that Fzd N-termini are highly conserved suggests that it is likely that glycosylation regulates all Fzd receptors in a similar manner. Nevertheless, this remains to be determined and other PTM modifications in the CNS remain to be identified. Given the important role of other PTMs in regulating the localisation and function of Fzd receptors in other tissues, future studies will shed light into the molecular mechanisms that control the localisation of these receptors to specific plasma membrane domains and how local activation of Wnt signalling in specific neuronal/cellular compartments is achieved and how they influence neuronal connectivity in the nervous system.

## Author Contributions

PP-V and PS designed the outline of the review and wrote the manuscript. PP-V created the figures. Both authors contributed to the article and approved the submitted version.

## Conflict of Interest

The authors declare that the research was conducted in the absence of any commercial or financial relationships that could be construed as a potential conflict of interest.

## Publisher’s Note

All claims expressed in this article are solely those of the authors and do not necessarily represent those of their affiliated organizations, or those of the publisher, the editors and the reviewers. Any product that may be evaluated in this article, or claim that may be made by its manufacturer, is not guaranteed or endorsed by the publisher.
